# Stability of Antibacterial Silver Carboxylate Complexes against *Staphylococcus epidermidis* and Their Cytotoxic Effects

**DOI:** 10.3390/molecules23071629

**Published:** 2018-07-04

**Authors:** Maialen Aldabaldetrecu, Laura Tamayo, Romina Alarcon, Mariana Walter, Edison Salas-Huenuleo, Marcelo J. Kogan, Juan Guerrero, Maritza Paez, Manuel I. Azócar

**Affiliations:** 1 Faculty of Chemistry and Biology, University of Santiago de Chile, Av. Bernardo Ó Higgins 3363, Casilla 40, Correo 33, Estación Central, 9170022 Santiago, Chile; maialen.aldabaldetrecu@usach.cl (M.A.); romina.alarcon@usach.cl (R.A.); mariana.walter@usach.cl (M.W.); juan.guerrero@usach.cl (J.G.); maritza.paez@usach.cl (M.P.); 2 Facultad de Ingeniería, Instituto de Ciencias Químicas Aplicadas, Polymers and Macromolecules Center, Universidad Autónoma de Chile, El Llano Subercaseaux 2801, San Miguel, 9170022 Santiago, Chile; lauratamayo26@gmail.com; 3 Departamento de Química Farmacológica y Toxicológica, Facultad de Ciencias Químicas y Farmacéuticas, Advanced Center for Chronic Diseases (ACCDiS), Universidad de Chile, 9170022 Santiago, Chile; edison.salash@gmail.com (E.S.-H.); mkogan@ciq.uchile.cl (M.J.K.)

**Keywords:** silver antibacterial, *Staphylococcus epidermidis*, human ovarian cancer cell (A2780), murine fibroblast

## Abstract

The antibacterial effects against *Staphylococcus epidermidis* of five silver carboxylate complexes with anti-inflammatory ligands were studied in order to analyze and compare them in terms of stability (in solution and after exposure to UV light), and their antibacterial and morphological differences. Four effects of the Ag-complexes were evidenced by transmission electronic microscopy (TEM) and scanning electronic microscopy (SEM): DNA condensation, membrane disruption, shedding of cytoplasmic material and silver compound microcrystal penetration of bacteria. 5-Chlorosalicylic acid (5Cl) and sodium 4-aminosalicylate (4A) were the most effective ligands for synthesizing silver complexes with high levels of antibacterial activity. However, Ag-5Cl was the most stable against exposure UV light (365 nm). Cytotoxic effects were tested against two kinds of eukaryotic cells: murine fibroblast cells (T10 1/2) and human epithelial ovarian cancer cells (A2780). The main objective was to identify changes in their antibacterial properties associated with potential decomposition and the implications for clinical applications.

## 1. Introduction

The antibacterial applications of silver in medicine have been widely studied [1]. Besides applications as an antimicrobial agent, the biological activity is associated with high effectiveness, low toxicity and virtually no resistance of microorganisms to the presence of this metal [2]. Ag(I)-complexes act by various mechanisms against microorganisms, and their ligands play a role as metal carriers in biological structures like membranes, DNA, enzymes, and others [3]. However, antibacterial agents should have other properties to be good candidates for future medical applications, such as being light-insensitive (including to UV light), having thermal stability and good solubility [4,5,6]. Unfortunately, few studies have addressed the problem of stability and antibacterial activity simultaneously, nor additional applications for the ligands used in the preparation of the metal complexes [4,5,7].

In this paper, we present evidence of the stability of antibacterial Ag(I) carboxylate complexes in solution, and the influence of the nature of the Ag–OOC bond, in order to demonstrate the importance of ligands in these antibacterial complexes. Five silver complexes with anti-inflammatory ligands were tested for 4 weeks against *S. epidermidis*, which was chosen because of its resistance to antibiotics, including penicillin, amoxicillin, and methicillin, as well as its capacity to form biofilms on the plastic surfaces [8] of medical devices (catheters and prostheses) [9]. These kinds of studies can provide insights into understanding the biological activity of silver-based compounds as bacteria-inhibiting materials, with low cytotoxicity effects and chemical stability over time.

Silver compounds with anti-inflammatory ligands have recently been synthesized and their stability studied in the solid state with exposure to UV radiation. It has been found that the nature of the ligands, as well as the coordination number and geometry, can influence the stability in the context of exposure to light and temperature variations [5,7,10,11]. However, the stability of compounds in solution has not been widely studied, nor has the relationship between chemical properties and antibacterial activity over time. Moreover, these metal compounds may have lower toxicity and greater pharmaceutical effects than other kinds of ligands [12].

Differences were found among the compounds in antibacterial activity against *S. epidermidis*, with the level of antibacterial activity remaining stable in some compounds, at least for 21 days, while it decreased in others. The effects of the compounds on cell viability were tested with the MTT assay (a colorimetric assay for assessing metabolic cell activity) against two eukaryotic cell lines: T10 1/2 murine fibroblasts and A2780 human ovarian cancer cells.

## 2. Results

### 2.1. Stability of Silver Complexes

**Figure 1:**
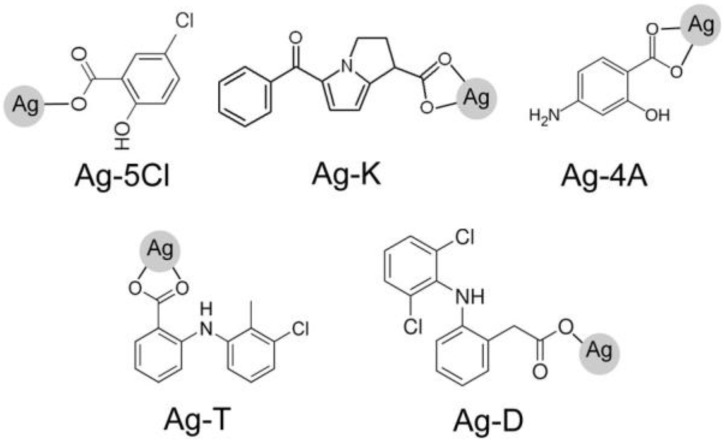
Anti-inflammatory ligands used in the synthesis of silver compounds: Ag-5Cl, Ag-K, Ag-4A, Ag-T, Ag-D, tolfenamic acid (T), ketorolac tris salt (K), sodium diclofenac (D), 5-chlorosalicylic acid (5Cl), and sodium 4-aminosalicylate (4A).

The silver(I) complexes (Ag-T, Ag-K, Ag-D, Ag-5Cl, and Ag-4A) were obtained in the form of white powders, which are slightly soluble in water [10], soluble in DMSO and insoluble in organic solvents. The structures feature silver ions coordinated with the ligand carboxylic acid groups [13,14] (see Figure 1).

Their stability with exposure to air and light varies, with the order of the most to the least stable being Ag-T = Ag-5Cl > Ag-4A > Ag-D > Ag-K. Ag-T and Ag-5Cl are the most stable over time but Ag-K decomposes with exposure to light and air, and at certain temperatures [10].

**Figure 2:**
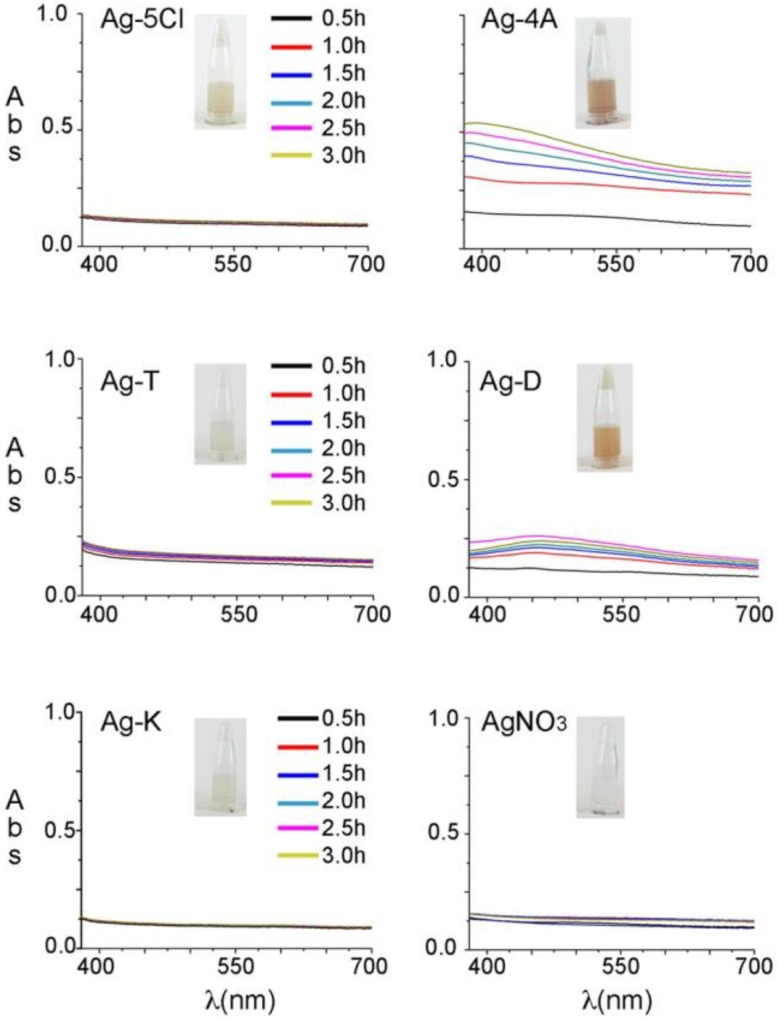
Absorption spectra of the silver solutions over time. Samples were irradiated at 365 nm for 3 h.

Before evaluating their antibacterial stability against *S. epidermidis*, the silver compounds were studied in an aqueous solution (2% DMSO) and their photophysical stability was evaluated after UV light irradiation. Other organic solvents were tested, but the silver compounds were insoluble or only slightly soluble in those. The solutions were irradiated at 365 nm (7 watts) for 3 h and the spectra analyzed every 30 min in a range of 350–700 nm to register color changes in the visible region. Figure 2 shows the evolution of the visible spectra over time and the effects of exposure to UV light. Ag-5Cl, Ag-T, Ag-K and AgNO_3_ did not show any changes in color over time, and remained colorless in solution/suspension. Similarly, there were no evident changes when the samples were exposed to sunlight for 4 weeks. In contrast, Ag-4A and, Ag-D turned from colorless to brown after one hour of exposure to UV light (365 nm), or 3 days of exposure to sunlight.

When the pH of a solution rises to 8 or over, silver ions react in solution and precipitate as AgOH and Ag_2_O [15]. The pH levels in all the solutions is in the range of 4 to 5, therefore, the formation of new insoluble silver species can be ruled out, but when the anti-inflammatory ligands are exposed to light, 4A and D (4-aminosalicylate and diclofenac) decompose to give a brown solution. The characteristics of diclofenac decomposition in water with exposure to UVA, UVC and natural sunlight have been described before [16]. Decomposition in the case of 4-aminosalicylate could be the result of decarboxylation in an acidic medium. *m*-Aminophenol may be the main compound formed in the solution because of the pH level of 5.0 when the Ag-4A is dissolved in water [17].

### 2.2. Antibacterial Experiments

**Figure 3:**
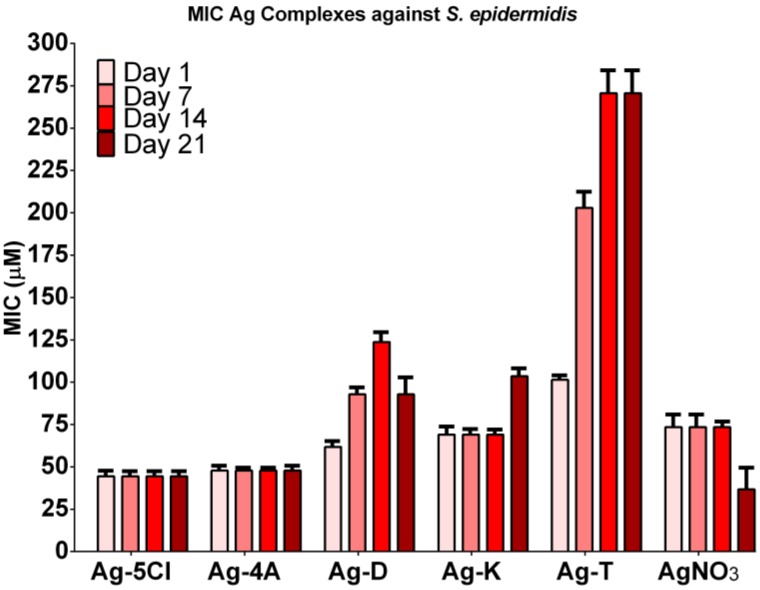
Antibacterial activity of Ag-complexes and AgNO3 against *S. epidermidis* over 4 weeks. Free ligand did not show antibacterial activity (MICs > 200 µg/mL).

The antibacterial stability of the silver compounds against *S. epidermidis* was tested over four weeks. Figure 3 shows the results. Ag-4A and Ag-5Cl were the most effective (although Ag-4A decomposes with exposure to air and light) and maintains its MIC values (45 µM) for at least four weeks. The antibacterial activity of Ag-K requires a higher dose. Inconveniently its effectiveness decreases by the fourth week by more than 50%. In the case of ketorolac, studies have reported decomposition by decarboxylation, followed by autoxidation and pH-dependent kinetics [18]. It has also been determined that ketorolac rapidly decomposes in aqueous solutions and ethanol under UV light owing to the production of four types of products and CO_2_ [19]. Given the characteristics of ketorolac, the decomposition of this ligand could reduce the antibacterial effect of silver compounds in the context of a pH from 4 to 5 and an extended period of exposure to sunlight.

MIC for Ag-D increased over time (62, 93 and 124 µM), although it decreased in the last week (93 µM), which may reflect activity over time, and finally an increase in toxicity caused by the decomposition of the diclofenac ligand [16]. The most significant decrease in activity was with Ag-T, from 101.4 to 271 µM. The MIC of this compound against *S. epidermidis* increased by 250%, although it was more stable than the other compounds. Free ligands did not show antibacterial activity even at 1000 µM, therefore the antibacterial activity depends primarily on the silver ion. AgNO_3_ was used as a control and as a free Ag^+^ ion in water. In a slightly acidic solution, decomposition to silver oxide and change in color were not observed, as was expected. Under the same conditions, Ag-4A and Ag-5Cl showed a potential synergic effect in MIC values, demonstrating the effect of ligands on the antibacterial activity of the silver compounds [3].

As our group has reported, Ag-5Cl shows a high degree of stability against high energy UV light in the solid state. The tests in solution also show that AgCl is stable with exposure to UV light. These characteristics are related to the phenolic ionization constant of AgCl (pKa: 13.34) and the ionization constant of the carboxyl group (pKa: 4.85), which contribute greatly to the stability of the metal compounds, as has been found for metals like iron and aluminum [20]. Salicylic acid and its derivatives have been used to provide antibacterial properties to different types of materials. The results have shown a remarkable antibacterial activity of 5-chlorosalicylic acid against *Escherichia coli*. [21] Therefore, this acid (ligand) could contribute to the antibacterial properties of silver complexes.

Recently, a polymeric silver(I) complex with diclofenac [12] was tested against a wide range of microorganisms, demonstrating a broad spectrum of antibacterial activity for this type of compound, even with similar values 16–64 (µg/mL) to those obtained in this study. But as usual, trials were performed with fresh solutions, or without considering antibacterial stability over time. However, this information is important because of the high sensitivity of silver compounds to exposure to light, air, and water [4,5,7,15].

Silver ions produce silver oxides as a precipitate in water, which change the solution from being colorless to having brownish black color and react with protons (H^+^) to generating free Ag^+^ [22]. Because ligands stabilize the metallic center, the advantages of using them to obtain Ag compounds include control over ion release and maintaining antibacterial efficacy [2,3,4,5,23].

### 2.3. Viability Test

The rate of release of Ag^+^ ions from carboxylic compounds may be key to explaining the stability and cytotoxic effects of antibacterial activity. After in vivo studies of silver salts (I) and silver sulfadiazine, Fox and Modak [23] concluded that compounds with more covalent bonds would have slower and more sustained release of Ag ions. Viability assays correlated the antibacterial activity of the compounds over time.

**Figure 4:**
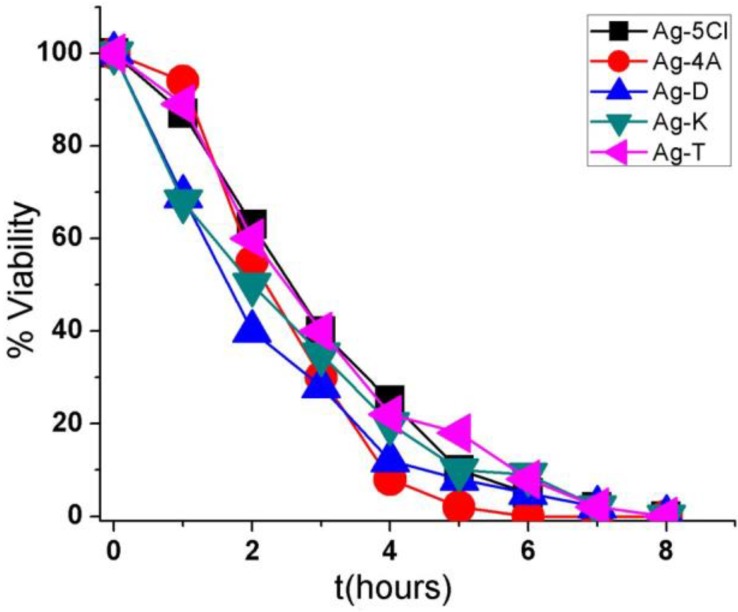
Viability (percentage of CFU/mL) as a function of time (hours) for the five compounds against the bacterium *Staphylococcus epidermidis*.

Figure 4 shows the results obtained with *S. epidermidis* after exposure to one of the five silver compounds in a concentration 30 µg/mL. There was a 100% reduction in bacterial viability after 6 h of exposure to Ag-4A and 8 h to Ag-D, Ag-5Cl, Ag-T, and Ag-K. Ag-4A may be more efficient over time because of its higher silver content (41.46%). In contrast, Ag-5Cl, with similar silver content (38.60%), required more time to eliminate the entire bacterial population, which could be because of a more covalent bond between silver ion and the carboxylate group of the ligand [10], greater stability in solution, and slower release of silver ions.

Ag-D, Ag-T, and Ag-K have similar silver ion contents and effectiveness over time, although the characteristics of their ionic bonds are different. These differences may be associated with Ag+ ion release or with the fact that these compounds act without ion dissociation, or they may be based on mechanisms in the cells.

The effectiveness and antibacterial stability of silver complexes may be important factors for the design of new materials such as medical implants, dental photopolymers, and catheters. For these reasons, it is important to know their activity in time and stability as additives. When these results are compared to those from other publications in which similar experimental conditions have been used, it is evident that *S. epidermidis* is more resistant than *Escherichia coli* and *Staphylococcus aureus*, given that more time is required in trials to reduce the population [12,22,23].

### 2.4. TEM

**Figure 5:**
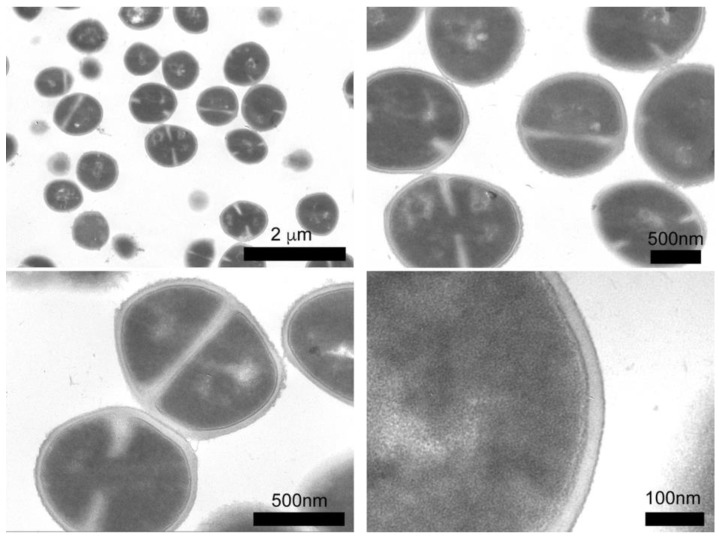
Cross sections of *S. epidermidis* (control sample) by TEM.

**Figure 6:**
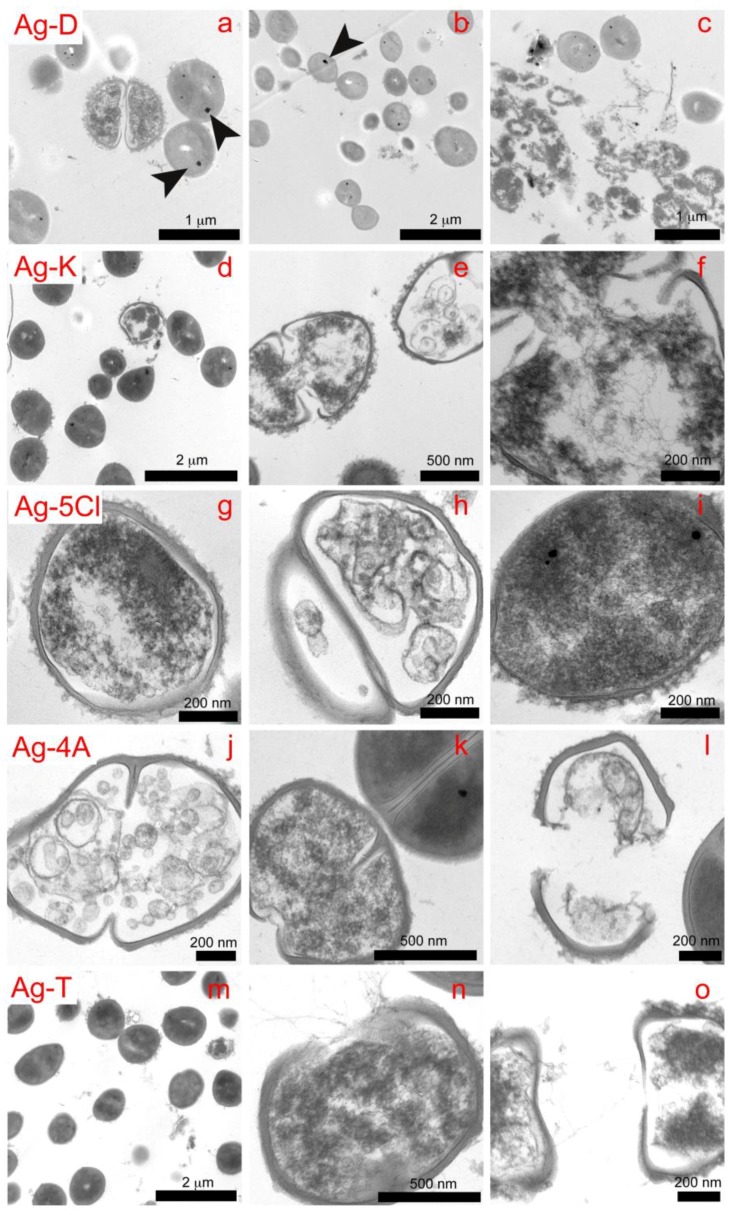
Cross sections showing the effects on *S. epidermidis* of all the compounds (Ag-D, Ag-T, Ag-5Cl, Ag-4A, and Ag-T) after 45 min of exposure.

We employed TEM microscopy to understand the contribution of the silver compounds in the antibacterial mechanism. This technique allows for studying the morphological and structural changes caused by compounds in bacteria. The control sample (see Figure 5) shows the intra-, and extracellular morphology of *S. epidermidis*, with 90% of the bacteria showing cell division and spherical morphology, as well as well-defined cell membranes and walls. The interior of the bacteria is a homogeneous gray color, which reflects a uniform cytoplasm density. For the assay, *S. epidermidis* was exposed to the compounds for 3 h. The effects on the bacteria caused by the compounds are shown in Figure 6, in which the micrographs show cross sections of the bacteria, this allowed for analyzing changes in walls and interior of bacterial cells.

With Ag-D, we observed loss of bacterial lysis, wall thickness and cytoplasmic material (Figures 6a–c). In addition, black spots appeared in the bacteria, which could be microcrystals of the respective compounds. This would demonstrate the ability of compounds to penetrate cell walls. In the upper right image (Figure 6c), contamination of *E. coli* was observed, which appears to be more sensitive to wall rupture and condensation of cytoplasmic material than is *S. epidermidis*. This concurs with the results of other viability studies [24,25,26,27], which have shown higher mortality rates for *E. coli*.

The percentage of bacteria in replication was lower with Ag-K than with the control, indicating that Ag-K interferes with the mechanisms of DNA replication or condensation [28]. This effect can be seen in the image on the right (Figure 6f), with condensed DNA strands that demonstrate antibacterial action in the cell [29].

Bacterial lysis was not observed with Ag-5Cl, but damage to the interior of the cell was. The image on the left (Figure 6g) shows how the cell wall loses thickness and definition, as well as condensation of cytoplasmic material, which disables or interrupts vital functions like enzymatic processes. The image on the right (Figure 6i) shows several black spots that may be associated with the penetration of microcrystals, which, to the best of our knowledge has not been evidenced to date for silver compounds, although it has been observed with silver nanoparticles [26].

The three main effects observed with Ag-4A (Figure 6j–l) are DNA condensation, loss in the definition of the cytoplasmic wall and membrane lysis. Black spots (Figure 6k), representing microcrystals of compound, appear again in the bacteria.

The bacterial population in the mitosis phase (Figure 6m) decreased after exposure to Ag-T, which evidences the interaction of the compound with DNA. This interaction is shown in the image (Figure 6n–o), in which the DNA is condensed into the form of strands. Again, lysis, loss of consistency and definition of cell walls, and loss of cytoplasmic material is observed (Figure 6n–o). These results show that the compounds interact with cell walls and membrane, proteins and eventually DNA. Their effectiveness is associated with several mechanisms of simultaneous action.

A study of the role of Ag+ in destabilizing *S. epidermidis* adhesion to biofilms showed that silver ions in ppb concentrations are ineffective against cells residing in biofilms. No significant changes in viability were observed, even after increasing the contact time. Silver ions are probably trapped in the biofilm matrix, making it ineffective to penetrate the deeper core of the biofilm matrix where the bulk of the bacterial cells are usually present [30,31].

Silver ions also interact with molecules in the EPSs [32,33] and even with a large number of halides and other ions, like Cl^−^, HCO_3_^−^, and CO_3_^−2^, and protein anions [34,35] to produce insoluble silver salt, which causes silver ion inactivity. This also implies that low concentrations of silver ions are unsuitable for the treatment of biofilm infections. Consequently, ligands can play a role as silver ion carriers in bacteria and prevent interaction with other molecules in the external environment.

### 2.5. Scanning Electron Microscopy (SEM)

**Figure 7:**
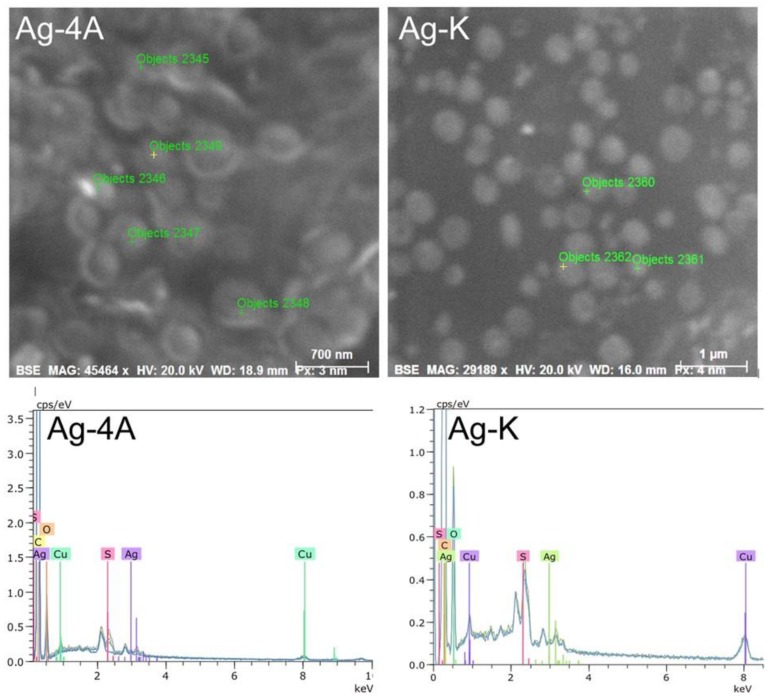
Black spots observed in TEM and analyzed by EDS-SEM for composition.

The black spots observed with transmission electron microscopy (TEM) were microanalyzed by scanning electron microscopy and EDS to determine their composition. The results obtained for the Ag-4A and Ag-K compounds are shown as an example in Figure 7.

Figure 7 shows the composition and ion content of each point. The black spots in the bacteria are silver, which was detected at every point that was analyzed. The largest amount was 1.04%, found at point 2356, as shown in Table 1.

Ag-D, Ag-5Cl, and Ag-T also revealed the presence of up to 1.04% silver in all the objects analyzed. This technique demonstrated the presence of silver in the bacteria in the form of particles or crystals of the complexes.

### 2.6. Cell Viability Assay

**Figure 8:**
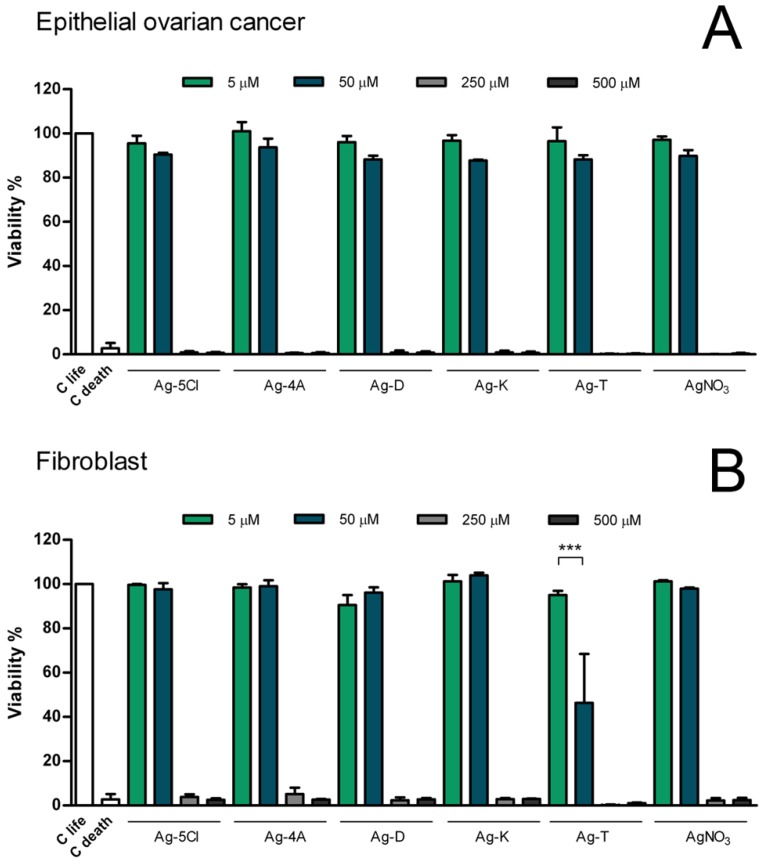
Evaluation of cellular viability in human cell lines. The viability of fibroblasts (**A**) and epithelial ovarian cancer cells (**B**), were evaluated after 24 h exposure to Ag coordination compounds. Results are presented as mean standard deviation of three independent experiments. For all treatment at 5 and 50 µM no statistical significance was found compared with control. *** = *p* < 0.001.

Given the improved antibacterial activities of Ag coordination compounds and the differences in MIC values, we sought to determine if silver compounds have potential medical applications. We assayed the cytotoxic effects (Figure 8) of these compound on eukaryotic cells using mitochondrial activity of metabolically active cells as a linear indicator of cell viability. The assays employed murine fibroblast T10 1/2 cells and human epithelial cancer cells.

Cells of both cell lines died with all the compounds at concentrations of 500 and 250 µM, but cell viability was not affected at concentrations of 50 and 5 µM, with the exception of the compound Ag-T, which reduced the viability of fibroblast cells by 50% at a concentration of 50 µM. However, in comparison with other tolfenamic metals (Cu, Co, and Zn), the silver compounds are less toxic [36]. These results can be explained by reference to other investigations that have shown the cytotoxic effects of tolfenamic acid [37] on the proliferation of nasopharyngeal cancer cell lines and a normal keratinocyte cell line.

In vitro assays using mammalian cell cultures have provided valuable information about the toxicity of compounds. Determining the basal cytotoxicity of silver compounds is an important step in toxicity studies. Another important step will be studying the decomposition of Ag compounds and the implications in mammalian cells, but it is necessary to know the behaviors of these compounds in initial states. In typical experiments, such as MTT assays, cultures of various types of human cells can be used to estimate the toxicity of silver compounds and their potential applications.

In this context, using the compound Ag-T as an antibacterial agent implies employing a higher dose (as evidenced by the MIC results), which could have undesired effects on cytotoxic activity and disfavors its clinical use. In contrast, Ag-5Cl shows greater chemical stability and requires lower doses over time. These doses remain sufficiently low with respect to the viability tests, which require concentrations close to 250 uM to reduce 100% of cell viability.

## 3. Materials and Methods

### 3.1. Synthesis and Stability of Silver Complexes

Five silver(I) complexes with anti-inflammatory ligands (tolfenamic acid (T), ketorolac tris salt (K), sodium diclofenac (D), 5-chlorosalicylic acid (5Cl) and sodium 4-aminosalicylate (4A)) had been synthesized and characterized previously by our group, as reported. However, the biological properties had not been studied [10,13,14]. Synthetic procedures for all complexes involved straightforward mixing of previously treated ligands with NH_3_ at pH 8.0 in an ethanol/aqueous solution with AgNO_3_ in aqueous solution, Ag^+^: ligand = 1:1 M ratio. The syntheses were carried out under constant stirring for 15 min at room temperature. The insoluble products were filtered off, washed with cold water and ethanol, and dried in vacuo over calcium chloride at room temperature. Purity was confirmed by elemental analysis and NMR spectra [10]. Due to the light sensitivity of the Ag(I) complexes, it was important to evaluate their behavior in solution.

The stability of the silver complexes was studied in aqueous solution (2% DMSO) and their photophysical stability was evaluated after UV light irradiation for 3 h at 365 nm (7 watts). The absorption spectra were recorded every 30 min in a range of 350–700 nm. The objective was to record color changes associated with the decomposition of complexes.

### 3.2. Antibacterial Activity

The antimicrobial activity of the compounds was determined with the broth microdi-lution method, as recommended by the National Committee for Clinical Laboratory Standards. Minimum inhibitory concentrations (MICs) of the compounds were tested against *S. epidermidis* (ATCC 12228). Stock solutions of the silver compounds in DMSO (2%) were diluted in broth (Muller-Hinton) to final concentrations of 3–200 µg/mL. Free ligands were also tested in quantities from 5 to 200 µg/mL. No antibacterial activity was observed, even with the highest concentration. AgNO_3_ was also tested and used as a control.

Minimum inhibitory concentrations (MIC) were determined after 24 h of incubating 10^5^ CFU/mL of the respective compound concentrations at 37 °C. Samples of 300 µL were transferred (in sextuplet) to 96-well immunoplates (ELISA plates) and incubated for 24 h at 37 °C, with continuous shaking. The growth inhibiting effect was measured using optical density at 600 nm (Labsystem Multiskan GO, Thermos), and recorded hourly for 24 h. The MIC was the lowest concentration at which bacterial growth was inhibited. The stability of the antibacterial activity of the silver compounds over time was tested over 4 weeks. The stock solutions of the silver compounds were kept at room temperature and exposed to sunlight throughout the period. Samples were taken weekly and diluted to be evaluated against *S. epidermidis*, as described above.

### 3.3. Morphological Changes by TEM/SEM Images

An aliquot of 3 mL of bacterial solution, standardized to 10^5^ CFU/mL, was incubated for 45 min with exposure to the silver compounds. Following incubation, the samples were washed with buffer and centrifuged at 655 g for 12 min to obtain bacterial pellets.

The pellets were fixed by exposure to a glutaraldehyde solution at 2.5% in cacodylate buffer for 30 min, followed by dehydration of the bacteria using 50%, 60%, 70%, 90%, and 100% ethanol/cacodylate buffer solutions. The bacteria were finally embedded in epoxy resin and polymerized in an oven at 60 °C for 24 h. The samples were sectioned into 100-nm-thick slices, deposited on a copper grid (400 mesh) and observed in by TEM using a Philips Tecnai 12 instrument [38]. *S. epidermidis* was also analyzed by scanning electron microscopy (SEM) on a Teslan Vega 3 Quantax 400 model instrument equipped with a Bruker probe to observe morphological changes and the presence of silver in the bacteria.

### 3.4. Cell Viability Assay

A tetrazolium salt reduction assay (CellTiter 96^®^) established a linear relationship between the number of viable cells and the absorbance of the reaction product. The assay involved seeding a T10 1/2 murine fibroblast cell line and a human ovarian cancer cell line with Dulbecco’s Modified Eagle’s Medium/Nutrient Mix F12 (DMEM/F12) on 96-well plates at a density of 5000 cells/well. The cells were then treated with Ag coordination compounds (Ag-5Cl, Ag-4A, Ag-D, Ag-K, and Ag-T), and AgNO_3_ as a control, and all dissolved in mili-Q water from a DMSO aliquot, at concentrations of 5, 50, 250 and 500 µM. Cell viability was measured in triplicate after 24 h of incubation at 37 C, with 5% CO_2_. Absorbance was recorded at 490 nm on a Multiscan reader. The data was statistically analyzed with a Kruskal-Wallis test and a Dunn’s post-test, with a statistical significance at *p* < 0.05, using the GraphPad Prism 5.03 program (GraphPad software, Inc., San Diego, CA, USA).

## 4. Conclusions

Our results suggest the antibacterial activity and stability of silver compounds over time depend on the nature of the ligands and their potential decomposition. Ag-5Cl is the best additive for medical applications because it is the most stable in solution and with exposure to light, and as an antibacterial agent due to the low required dose. Tolfenamic, ketorolac, diclofenac and 4-aminosalicylate decompose in aqueous solutions and with exposure to light, with significant loss in some cases of their antibacterial activity.

*S. epidermidis*, which is associated with biofilms on plastic surfaces, was affected in different ways by interaction with the five silver compounds, which indicate their antibacterial properties: membrane disruption, shedding cytoplasmic material, penetration of microcrystals, and possible condensation of DNA, which prevents DNA from replicating and cells from reproducing.

Silver compounds were not cytotoxic below concentrations of 50 M, with the exception of Ag-T, which reduced the viability of fibroblast cells by 50%. The results indicate that silver compounds have low toxicity, although it is necessary to study toxic effects associated with decomposition. In conclusion, this study provides insights into understanding the biological activity of silver-based compounds as bacteria-inhibiting materials, with low cytotoxic effects and chemical stability over time.

## Figures and Tables

**Table 1:** SEM-EDS elemental composition of the elements present in the analyzed points.

Points	Elements				
	C	O	S	Cu	Ag
2356	80.15	16.36	0.24	2.21	1.04
2357	80.86	15.58	0.56	1.69	0.31
2358	80.96	16.99	0.34	1.53	0.19
2359	81.59	16.00	0.41	1.72	0.27
